# Clinical determinants for fatality of 44,672 patients with COVID-19

**DOI:** 10.1186/s13054-020-02902-w

**Published:** 2020-04-28

**Authors:** Guangtong Deng, Mingzhu Yin, Xiang Chen, Furong Zeng

**Affiliations:** 1 The Department of Dermatology, Xiangya Hospital, Central South University, 87 Xiangya Road, Changsha, 410008 China; 2 Hunan Key Laboratory of Skin Cancer and Psoriasis, Changsha, China; 3 Hunan Engineering Research Center of Skin Health and Disease, Changsha, China; 4 National Clinical Research Center for Geriatric Disorders, Xiangya Hospital, Central South University, Changsha, 410008 Hunan China

Using meta-analysis to explore clinical determinants for death of COVID-19 patients has been a problem due to insufficient sample size and overlapped cases [1]. In the study, we re-analyzed the largest confirmed case series reported publicly by the Chinese center for disease control and prevention (44,672 laboratory confirmed cases updated through February 11, 2020, [2]), to explore the clinical risk factors associated with death.

The basic characteristics between survivors and non-survivors with COVID-19 were presented in Table 1. Among a total of 44,672 patients with laboratory confirmation of SARS-CoV-2 infection, 1023 (2.3%) patients were dead as of February 11, 2020, the last day of follow-up. The fatality rate was increasing with ages and even up to 14.8% in patients aged above 80 years old (see Fig. 1a). The prevalence of COVID-19 between men and women was pretty close (51.4% vs. 48.6%), which is different from previous report (58.1% vs. 41.9%) [3]. Notably, the fatality rate of male patients was significantly higher than that of female patients (RR = 1.67, 95%CI = 1.47–1.89, *p* < 0.001) (see Fig. 1b). Furthermore, cardiovascular disease (RR = 6.75, 95%CI = 5.40–8.43, *p* < 0.001), hypertension (HR = 4.48, 95%CI = 3.69–5.45, *p* < 0.001), diabetes (RR = 4.43, 95%CI = 3.49–5.61, *p* < 0.001), respiratory disease (RR = 3.43, 95%CI = 2.42–4.87, *p* < 0.001), and cancers (RR = 2.926, 95%CI = 1.34–6.41, *p* = 0.006) were the risk factors for fatality of patients with COVID-19.

**Table 1:** Characteristics between survivors and non-survivors with COVID-19

Characteristics	Total (*n* = 44,672)	Non-survivors (*n* = 1023)	Survivors (*n* = 43,649)	Fatality (%)	RR (95%CI)	*p*
Age, *n* (%)						
0–	416 (0.9%)	0	416 (0.9%)	0		
10–	549 (1.2%)	1 (0.1%)	548 (1.3%)	0.2		
20–	3619 (8.1%)	7 (0.7%)	3612 (8.3%)	0.2		
30–	7600 (17.0%)	18 (1.8%)	7582 (17.4%)	0.2		
40–	8571 (19.2%)	38 (3.7%)	8533 (19.5%)	0.4		
50–	10,008 (22.4%)	130 (12.7%)	9878 (22.6%)	1.3		
60–	8583 (19.2%)	309 (30.2%)	8274 (19.0%)	3.6		
70–	3918 (8.8%)	312 (30.5%)	3606 (8.3%)	8.0		
≥ 80	1408 (3.2%)	208 (20.3%)	1200 (2.7%)	14.8		
Severity*, *n* (%)						
Mild/moderate	36,160 (80.9%)	0	36,160 (82.8%)	0		
Severe	6168 (13.8%)	0	6168 (14.1%)	0		
Critical	2087 (4.7%)	1023 (100%)	1064 (2.4%)	49.0		
Gender, *n* (%)						
Male	22,981 (51.4%)	653 (63.8%)	22,328 (51.2%)	2.8	1.67 (1.47–1.89)	< 0.001
Female	21,691 (48.6%)	370 (36.2%)	21,321 (48.8%)	1.7		
Comorbidity^#^, *n* (%)						
Hypertension	2683 (12.8%)	161 (39.7%)	2522 (12.3%)	6.0	4.48 (3.69–5.45)	< 0.001
Diabetes	1102 (5.3%)	80 (19.7%)	1022 (5.0%)	7.3	4.47 (3.49–5.61)	< 0.001
Cardiovascular disease	873 (4.2%)	92 (22.7%)	781 (3.8%)	10.5	6.75 (5.40–8.43)	< 0.001
Respiratory disease	511 (2.4%)	32 (7.9%)	479 (2.3%)	6.3	3.43 (2.42–4.87)	< 0.001
Cancer	107 (0.5%)	6 (1.5%)	101 (0.5%)	5.6	2.93 (1.34–6.41)	0.006

*Missing data (*n* = 257 in survivors group)

^#^Missing data (*n* = 617 in the non-survivors group, *n* = 23,073 in the survivors group)

**Fig. 1:**
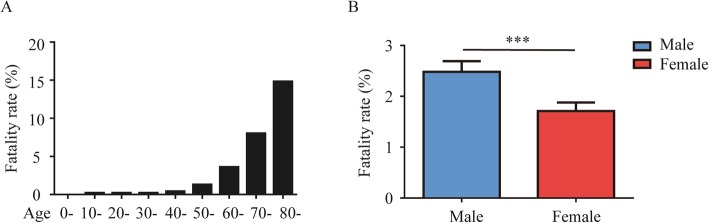
Fatality rate distribution of age (**a**) and gender (**b**). ***p < 0.001

In summary, we found that there was no difference in the prevalence of COVID-19 between men and women, but male patients had a nearly 1.7-fold higher risk of death than female patients. Moreover, we concluded that patients with comorbidities had a significantly high death risk. Admittedly, due to the unavailability of individual patient data, we could not exclude the influence of age on the conclusion because old patients were more likely to have the underlying comorbidities. We would like to provide a reminder to the physicians that more intensive surveillance or treatment should be considered for male patients and those with comorbidities. Further and larger studies are needed to validate the findings.

## Data Availability

Not applicable.
